# Host MiRNA responses during different waves of SARS-CoV-2: diagnostic implications of miR-19a-3p, miR-374b-5p, miR-15b-5p, and miR-320a-5p expression

**DOI:** 10.1186/s12879-026-12706-y

**Published:** 2026-02-16

**Authors:** Esraa S. AL-Aasar, Hoda Y. Abdallah, Ahmed S. Abu Zaid, Khaled M. Aboshanab, Mohamed A. Tantawy

**Affiliations:** 1 Department of Microbiology and Immunology, Faculty of Pharmacy, Ain Shams University, Cairo, 11566 Egypt; 2 AL-Ahrar Teaching Hospital, GOTHI, Zagazig, 44974 Egypt; 3 Department of Histology and Cell Biology, Faculty of Medicine, Suez Canal University, Ismailia, Egypt; 4 Hormones Department, Medical Research and Clinical Studies Institute, National Research Centre, 33-El Buhouth St, Dokki, Cairo, 12622 Egypt; 5 Faculty of Health Science, Higher Colleges of Technology, Sharjah, United Arab Emirates; 6 Center of Orthopaedics Research, and Translation Science (CORTS), Department of Orthopaedics and Rehabilitation, Pennsylvania State University, College of Medicine, Hershey, PA 17033 USA

**Keywords:** miRNAs, COVID-19, SARS-COV-2, Biomarker, Variant, Beta, Delta, Gene regulation, Severity

## Abstract

**Background:**

Given the pivotal roles of miRNAs as regulators of gene expression, circulating miRNAs offer promising non-invasive diagnostic and therapeutic avenues. The evolving landscape of SARS-CoV-2 variants poses challenges for traditional disease detection and control.

**Methods:**

To unveil the specific circulating miRNA profiles linked to COVID-19 and its correlation with severity levels, qPCR was conducted on five potential miRNAs in plasma samples obtained from 112 COVID-19 patients (comprising 58 severe, 50 moderate, and four mild cases) upon hospital admission during two waves (Beta and Delta variants) of the pandemic, alongside samples from 112 healthy controls. Moreover, correlations between miRNA expression and various clinical laboratory parameters were investigated.

**Results:**

Two miRNAs, including miR-19a-3p and miR-374b-5p, were upregulated, and two were downregulated (miR-15b-5p and miR-320a-5p), while the miR-423-5p expression was not changed in COVID-19 patients. ROC curve analysis demonstrated that miR-15b-5p and miR-320a-5p showed the highest specificity and sensitivity. In addition, significant correlations were found between miRNA expression and various laboratory parameters, suggesting that these miRNAs may serve as potential biomarkers for disease severity and progression. Further analysis during different COVID-19 waves and severity levels revealed consistent miRNA expression patterns among the two waves of infection, and most importantly, significant changes in expression among the mild/moderate versus severe infected patients, underscoring their diagnostic value upon different virus strain infections.

**Conclusion:**

While miRNAs do not replace RT-PCR for viral detection, they offer valuable diagnostic insights into host response and disease dynamics, and may enhance early screening, risk stratification, and monitoring in clinical settings. This study highlights the role of miRNAs as non-invasive biomarkers for the diagnosis and monitoring of COVID-19. In conclusion, the SARS-CoV-2 virus is constantly evolving and mutating. The data presented here explores the distinct role of circulating miRNAs within individuals affected by different variants of COVID-19, unveiling their promise as non-invasive diagnostic markers.

**Clinical trial number:**

Not applicable.

**Supplementary Information:**

The online version contains supplementary material available at 10.1186/s12879-026-12706-y.

## Background

WHO declared a global pandemic (SARS-CoV-2) on March 11, 2020, due to its widespread. About five years after the virus’s emergence, the WHO report states that over 777 million instances of SARS-CoV-2 infection have occurred to date [1]. Over 7 million people have died [2]. Global economic and health crises were precipitated by the emergence of SARS-CoV-2 in 2019. Despite significant advancements in understanding the mechanisms behind SARS-CoV-2 infection and therapies, the virus still ranks among the top three infectious illness-related causes of death worldwide [3]. The diverse molecular mechanisms underlying its evolution remain unclear. With an average length of 22 nucleotides and various forms, the miRNAs are categorized as a family of small noncoding RNAs that have been conserved throughout evolution [4]. They have well-established functions in many diseases and are described as the “micromanagers” of gene expression [5–8]. They can control one-third of all human protein-coding genes [9, 10]. The gene expression that codes for proteins to participate in vital pathological processes against infection, such as the immune system’s defense against viral infections, is primarily controlled by miRNAs [11, 12].

Non-coding RNAs (ncRNAs), essential to almost all biological activities, have a significant role in the pathophysiology of viruses [13]. A typical genomic structure is shared by SARS-CoV-2 and other beta coronaviruses [14]. The miRNAs were shown to be linked to COVID-19 outcomes, which may make it possible to measure the risk of catastrophic outcomes and create outcome prediction models, which would aid in individualized treatment plans that are as aggressive as possible for each patient. A small number of recent research studies have investigated circulating miRNAs that may be related to the severity and mortality of COVID-19 [15–18]. Numerous research studies have established that the SARS-CoV-2 genome encodes viral miRNAs that are essential for infectivity and can target several host genes [13] and are involved in viral pathogenesis [19, 20] and host cell defense mechanisms against SARS-CoV-2 [21, 22]. Competitive endogenous RNAs produced by SARS-CoV-2 may reduce the expression of miRNAs in host cells [23, 24], modulating SARS-CoV-2 replication [23, 25–27]. Apparently, the severe pathogenicity of the SARS-CoV-2 virus is attributed to several factors, most notably its aggressive replication within the lungs. This process is closely linked to hyperinflammation, cytokine storm resulting from immune dysregulation, and ultimately, tissue damage. Accordingly, this study focuses on five microRNAs (miRNAs) that are potentially associated with these four key pathological features: viral replication, hyperinflammation, cytokine storm, and tissue damage. MiR-15b-5p has been reported to suppress SARS-CoV-2 infection and proliferation by targeting the RNA-dependent RNA polymerase structure [28]. MiR-19a-3p modulates inflammatory cytokine production by inhibiting macrophage 1 activation and controlling the cytokine storm [29]; it is also highly upregulated in COVID-19 infection [30]. MiR-320a-5p is associated with tissue damage, endothelial dysfunction, and immune modulation, and is significantly downregulated in hospitalized COVID-19 patients [31]. MiR-374b-5p plays a role in T-cell regulation and cytokine signaling; its dysregulation may contribute to immune imbalance. Additionally, it regulates SARS-CoV-2 viral protein expression by inhibiting the C-FLIP protein [32]. MiR-423-5p is linked to cardiac stress and hypoxia, and is elevated in severe COVID-19 cases [33]. Global miRNA expression studies have consistently identified these five miRNAs as among the most dysregulated in the serum or plasma of COVID-19 patients [18, 30, 31, 33]. Based on these findings, the current study aims to address two key questions: 1- What are the expression patterns of these miRNAs in COVID-19 infection, and most importantly, across different waves of COVID-19? 2-How do these miRNAs correlate with clinical parameters (e.g., D-dimer, total leukocyte count [TLC], etc.) commonly used in the diagnosis and prognosis of COVID-19 infection? The selection of the miR-15b-5p, miR-320a-5p, miR-19a-3p, miR-374b-5p, and miR-423-5p was based on three criteria: (i) Strong prior evidence of direct interaction with SARS-CoV-2 genomic or host-response pathways, including apoptosis regulation, immune modulation, endothelial dysfunction, and inflammatory signaling; (ii) their consistent dysregulation across multiple independent studies, including in vitro, in vivo, and patient-based datasets, suggesting biological robustness; and (iii) their relevance to key COVID-19 pathophysiological processes such as cytokine signaling, coagulation abnormalities, and viral replication control. Therefore, the present study was designed to describe the signature of five circulating miRNAs (miR-15 b-5p, miR-320a-5p, miR-19a-3p, miR-374b-5p, and miR-423-5p), which were proven to have a pivotal role in COVID-19 infection at different levels in the plasma of COVID-19 patients compared to healthy individuals. Expression analysis of the respective miRNAs among hospitalized patients was examined and correlated with infection with varying variants of SARS-COV-2 to be used as potential biomarkers for diagnosing and monitoring the severity of SARS-CoV-2 infection.

## Methods

### Subjects’ selection and sample collection

A total of 112 COVID-19 patients (41 Males and 71 females) and 112 healthy individuals (54 Males and 58 females) were included in this study (Table 1). The number of cases (81 cases from wave II and 31 cases from wave III). Waves and variant names are declared according to the WHO and timing of sample collection, the second wave Beta variant (from Jan 2021- Feb 2021) [34] and third wave Delta variant (from April 2021 to Jun 2021) [35–37]. The Beta and Delta variants were confirmed by genomic sequencing as previously reported [38]. A total of 54 mild/Moderate and 58 severe COVID-19 cases were determined by the attending physician according to the WHO guidelines (https://www.who.int/westernpacific/emergencies/covid-19/information/asymptomatic-covid-19). Inclusion criteria included COVID-19 cases of both genders, aged 17–85, with a confirmed diagnosis of COVID-19 (by RT-PCR, serology [IgM positive at the time of sampling], chest CT, as well as signs and symptoms of SARS-CoV-2 infection). Healthy individuals of both genders, recruited from the hospital’s blood bank, were aged 17–81, had a negative RT-PCR test for SARS-CoV-2 infection, and were not previously infected with COVID-19, as documented in hospital records (negative SARS-CoV-2 IgG and IgM). According to hospital records, exclusion criteria were non-hospitalized cases and were not confirmed to have SARS-CoV-2 infection (negative RT-PCR, IgM negative, or both at the time of sampling). The peripheral blood (10 mL) was collected from lab residuals into EDTA anticoagulated tubes (Xinle K3EDTA tube) from healthy volunteers and COVID-19 patients at the time of diagnosis and stored at 4 °C until further processing (within two hours of collection). Blood samples were centrifuged at 3500 rpm for 15 min to obtain plasma. After separation, 100 µL cell-free plasma samples were added to a 500 µL Qiazol reagent and stored at -80 °C till further analysis. The local ethics committee of the Faculty of Pharmacy, Ain Shams University, approved the proposed project (ENREC-ASU-2021-17). The study was conducted in accordance with the Declaration of Helsinki guidelines and regulations. The unidentified blood samples were collected from “AL-Ahrar Teaching Hospital” laboratory residues during the routine checkup, with hospital approval of the study and waiving the patient’s informed consent, as the clinical samples were unidentified, and there was no contact with patients. The severity analyses (moderate vs. severe) were conducted using the combined cohort of both waves (Beta and Delta). This was done to ensure adequate statistical power, as separating severity groups by wave would have resulted in small subgroup sizes.

**Table 1:** Gender and age data of COVID-19 cases (*n* = 112) and healthy control (*n* = 112)

	Cases (*n* = 112)		Control (*n* = 112)	
	number	%	number	%
**Gender**				
male	41	36.6%	54	48.2%
female	71	63.4%	58	51.8%
**Age (Years)**				
Min-max	17.0–85.0		17.0–81.0	
Mean ± SD	59.07 ± 11.64		56.54 ± 11.72	
Median (IQR)	60.50 (52.50 − 67.0)		57.50(45.5–66.0)	

### miRNA expression analysis

Before RNA isolation, samples were thawed at room temp (15–25 °C) for 5 min or until they became completely homogenous. The circulating RNA was extracted from plasma samples using the Qiagen miRNeasy Kit (Qiagen, 217004, USA), following the protocol supplied by the manufacturer [39]. The cDNA synthesis was prepared according to the manufacturer’s recommendation using the miRNA-specific stem-loop RT primer [40, 41], followed by qPCR analysis using SYBR Green Assay. The qRT–PCR was performed according to the MIQE guidelines [42] Using the miScript SYBR Green PCR Master Mix (Qiagen, Cat No. 218073). This was followed by 10x miScript Primer Assay (Qiagen, Cat No. 218300) to amplify and detect the cDNA of the miR-15b-5p, miR-320a-5p, miR-374b-5p, miR-423-5p, and miR-19a-3p targets, with a reverse universal primer (Qiagen, Cat No. 218073). The tube was aliquoted and kept protected at -20 °C [40, 41]. The 10x miScript Primer Assay for RNU6B and SNORD (Qiagen, Cat No. 218300) was used as a reference to normalize the expression levels of miR-15b-5p, miR-320a-5p, miR-374b-5p, miR-423-5p, and miR-19a-3p in the plasma to correct any possible differences in RNA quantity or quality across samples. The tube was also aliquoted and kept protected at -20º C. Finally, StepOne™ Real-Time PCR System (Applied Biosystems, Foster City, USA) [43–45]. The quantification of miR-15b-p, miR-320a-5p, miR-374b-5p, miR-423-5p, and miR-19a-3p was determined using the relative quantification method to calculate differences in the expression level of a mature miRNA gene relative to a reference sample.

### Clinical lab analysis

During the blood sample collection, we collected the clinical lab investigation data for the infected subjects with COVID-19 with the approval of the hospital, while preserving the patient’s confidentiality. The lab data were as follows, CBC (HB, hematocrit, PLT count, TLC count), renal function tests (serum urea, creatinine), electrolytes (Na, K), arterial blood gas (pH, PCO_2_, HCO3, PO_2_), liver enzymes (bilirubin, albumin, ALT, AST), and inflammatory markers (INR, CRP, D-Dimer) (Table S1).

### Statistical analysis

The data were analyzed statistically using IBM SPSS software Package Version 20.0 (Armonk, NY: IBM Corp). Shapiro–Wilk and Kolmogorov–Smirnov tests were utilized to test the normality of data. The chi-square test, Mann-Whitney test, Student’s t-test, and Kruskal-Wallis test were applied where appropriate. Spearman’s rank test was employed for correlation analysis. To evaluate the diagnostic and prognostic value of the investigated miRNA, the receiver operating characteristic (ROC) area under the curve (AUC) was plotted.

## Results

### Circulating miRNA signature in COVID-19 patients’ plasma

**Fig. 1:**
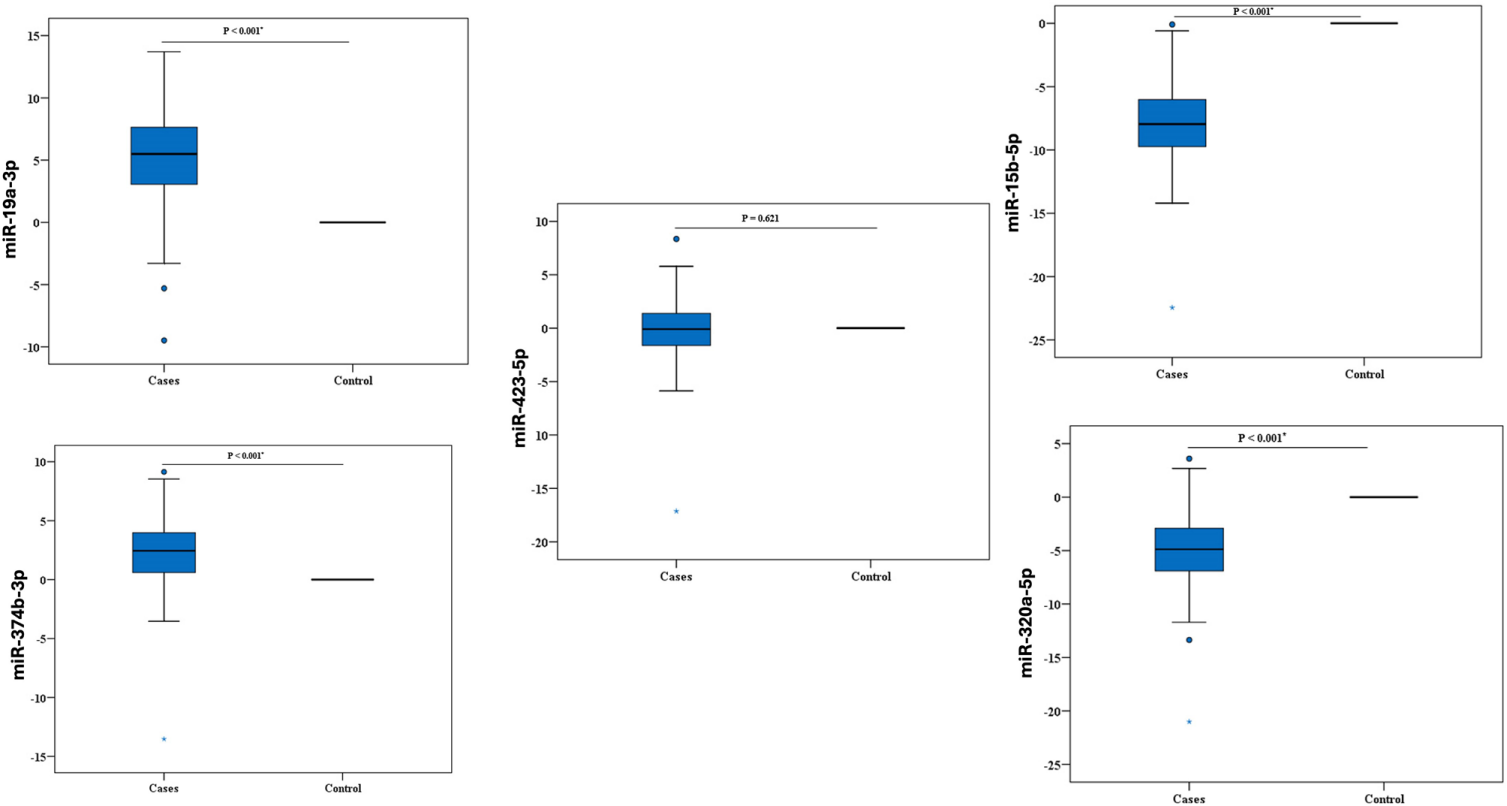
Differential expression of the five tested miRNAs among control (n=112) and COVID-19 cases (n=112) using quantitative RT-PCR

Using the StepOneTM Real-Time PCR System, qRT-PCR has been used to identify miRNAs that are differentially expressed in the plasma of infected patients (*n* = 112) in comparison to healthy individuals (*n* = 112). In the plasma of the COVID-19 patients, it was found that the expression of miR-15b-5p and miR-320a-5p were downregulated (7.96, and 4.88 fold change, respectively, compared to healthy control (*P value* < 0.001)), while the expression of miR-19a-3p, miR-374b-5p were upregulated (5.50, and 2.45 fold change, respectively, compared to healthy control (*P value* < 0.001)) and no difference in the expression of miR-423-5p (-0.09 fold change compared to health control (*p-value*, 0.621) was observed (Fig. 1). The statistical analysis of the obtained records of the five examined miRNAs is displayed in Table S2.

### Diagnostic accuracy of the selected plasma miRNAs

**Fig. 2:**
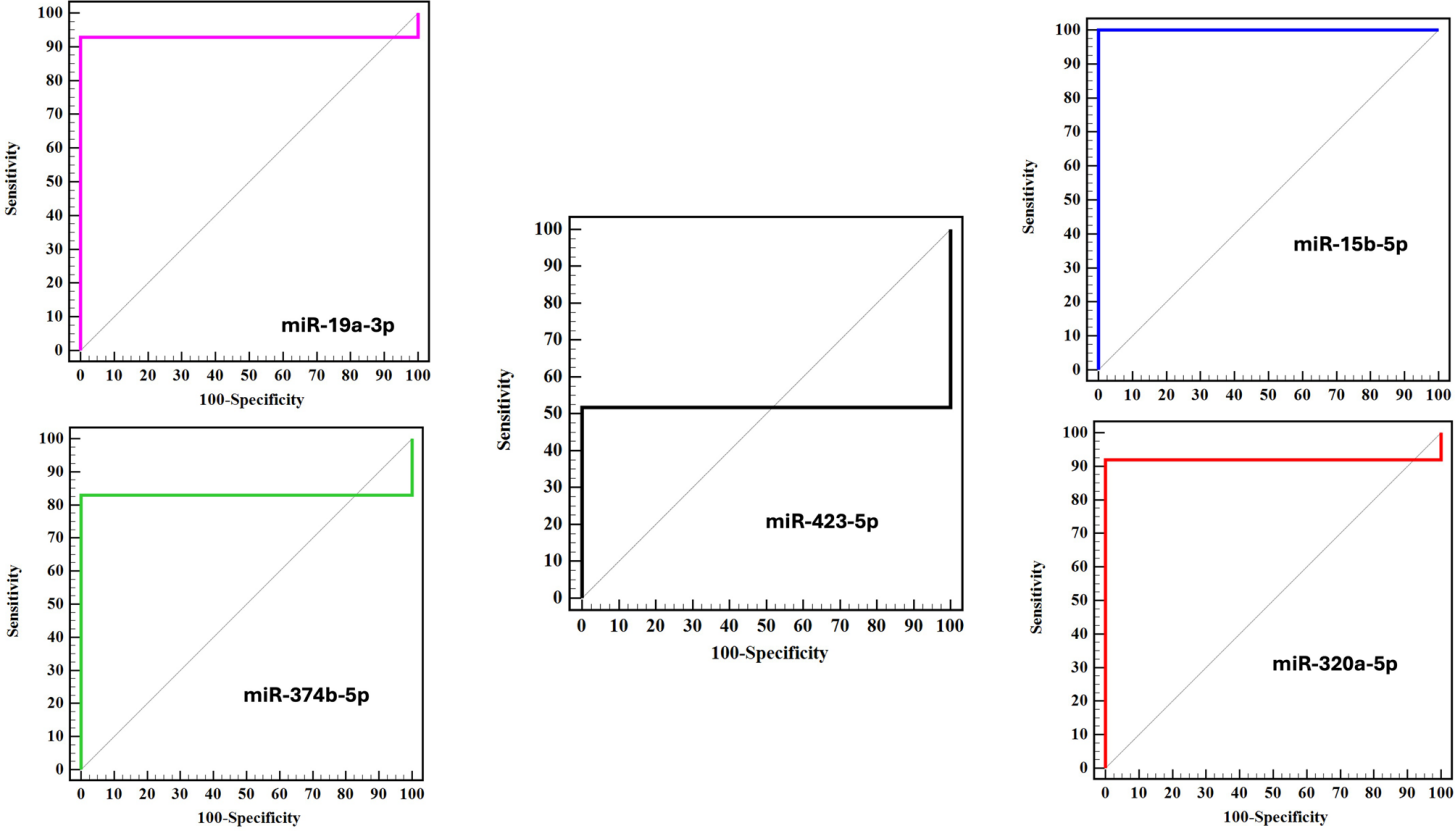
ROC curve analysis of the five tested miRNAs among control (n=112) and COVID-19 cases (n=112)

To investigate the diagnostic precision of the statistically significant and differentially expressed miRNAs, ROC curve analysis was utilized: miR-19a-3p, miR-15b-5p, miR-374b-5p, and miR-320a-5p (Fig. 2). As depicted in Fig. 2, the miR-320a-5p, miR-15b-5p, miR-19a-3p, and miR-374b-5p may function as prospective diagnostic biomarkers to differentiate healthy individuals from SARS-CoV-2-infected patients with an AUC of 0.920 (*P* < 0.001^*^), 1.000 (*P* < 0.001^*^), 0.929 (*P* < 0.001^*^), and 0.830 (*P* < 0.001^*^), respectively. At the cut-off value ≤ -0.13 for miR-320a-5p, the specificity and sensitivity were 100.0% and 91.96%, respectively. At the cut-off value ≤ -0.09 for miR-15b-5p, the specificity and sensitivity were 100% and 100%, respectively. At the cut-off value > 0 for miR-19a-3p, the specificity and sensitivity were 100% and 92.86%, respectively. At the cut-off value > 0 for miR-374b-5p, the specificity and the sensitivity were 100% and 83.04%, respectively (Table 2).

**Table 2:** Area under the curve (AUC) (95% confidence interval) for the examined MiRNAs

Marker	AUC	95% CI for AUC	*p*-value
miR-320a-5p	0.920	0.869–0.970	< 0.001^*^
miR-15b-5p	1.000	1.000–1.000	< 0.001^*^
miR-19a-3p	0.929	0.881–0.976	< 0.001^*^
miR-374b-5p	0.830	0.761–0.900	< 0.001^*^
miR-423-5p	0.518	0.425–0.610	0.644

### Circulating miRNA signatures in COVID-19 patients’ plasma obtained during different waves

**Fig. 3:**
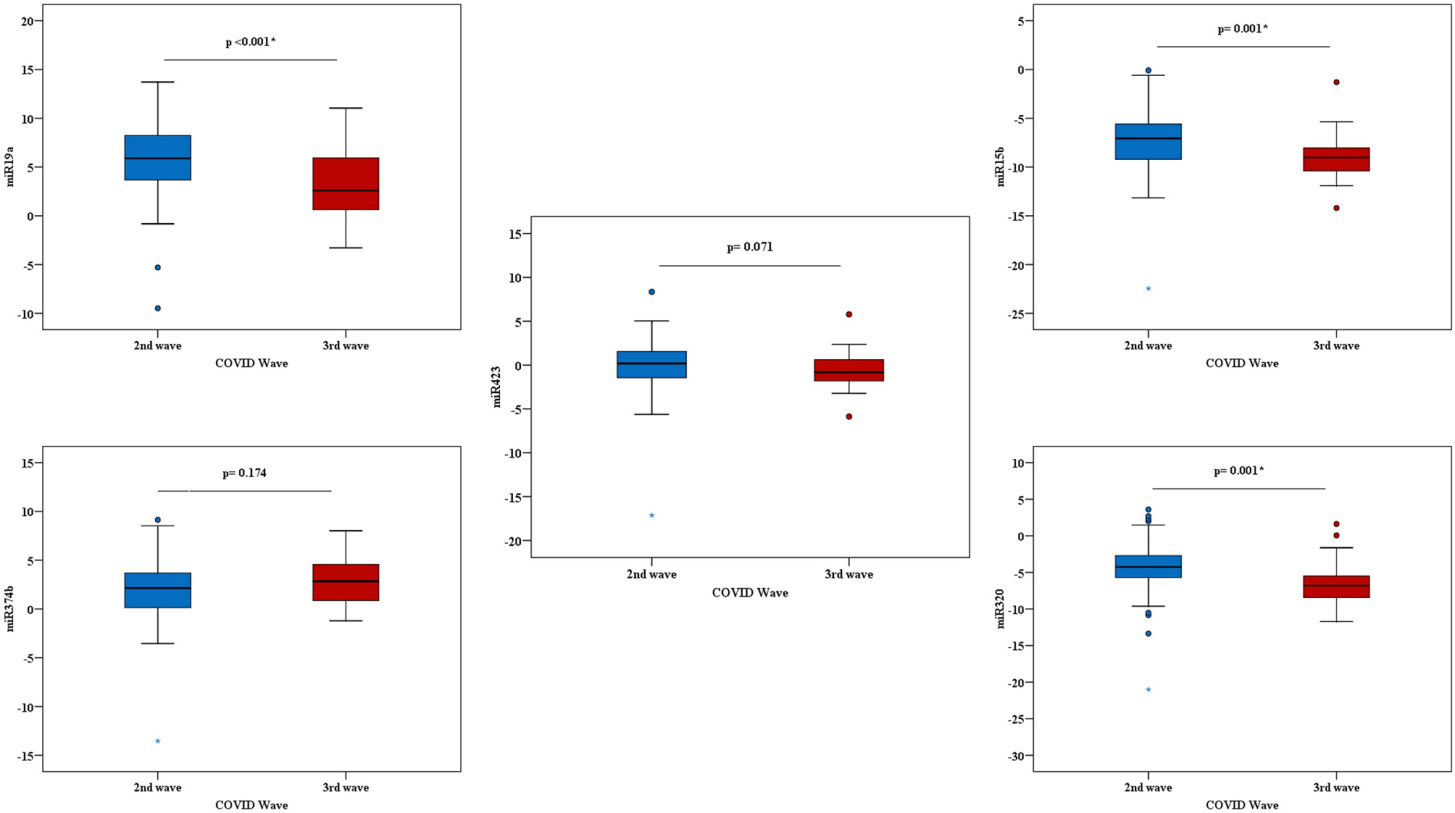
Differential expression of the five tested miRNAs of SARS-CoV-2-infected patients (2nd (n=81) versus 3rd (n=31) waves of SARS-CoV2 infections)

To further elucidate the expression pattern of the selected miRNA during different COVID-19 pandemic waves, plasma of infected patients collected during the second wave (beta variant) and third wave (delta variant) were collected. The qRT-PCR was performed on the five selected miRNAs, and three of them could differentiate between the two variant-infected groups (miR-320a-5p, miR-19a-3p, and miR-15b-5p). MIR-320a-5p expression during the third wave was statistically downregulated compared to the second wave (*P* = 0.001^*^). And MIR-15b-5p expression during the third wave was statistically downregulated compared to the second wave (*P* = 0.001^*^). MiR-19a-3p expression during the second wave was statistically upregulated compared to the third wave (*P* < 0.001^*^). While miR-374b-5p and miR-423-5p could not differentiate between the two infected variants (beta and delta) cases (*p-value*, 0.174, 0.071) respectively, as depicted in Fig. 3. the statistical analysis of the obtained records of the five examined miRNAs concerning the 2nd (*n* = 81) and 3rd (*n* = 31) waves, as compared to the control group (*n* = 112), is shown in Table S3.

### Diagnostic accuracy of the selected plasma miRNAs during different waves

**Fig. 4:**
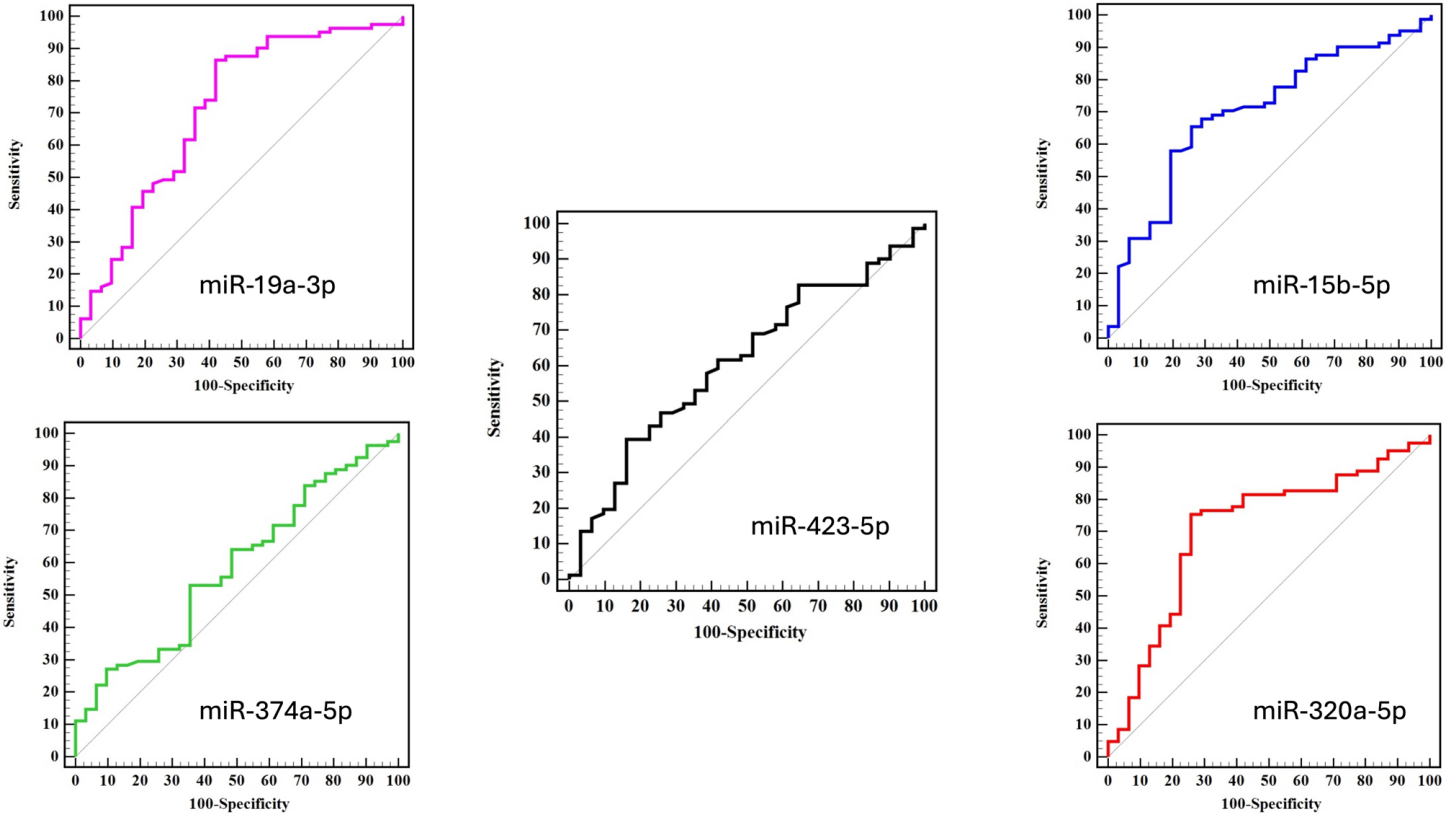
ROC curve analysis of the five tested miRNAs among (2nd (n=81) Versus 3rd (n=31) waves of SARS-CoV2 infections)

To investigate the diagnostic precision of the statistically significant and differentially expressed miRNAs between different variants, ROC curve analysis was utilized: miR-320a-5p, miR-15b-5p, miR-19a-3p, in (Fig. 4). As depicted in Fig. 4, the miR-320a-5p, miR-15b-5p, and miR-19a-3p may function as prospective diagnostic biomarkers to differentiate SARS-CoV-2 beta variant infected patients from SARS-CoV-2- delta variant infected patients with an AUC (the areas under the ROC curve) of 0.708 (*P* = 0.001^*^), 0.700 (*P* = 0.001^*^), and 0.715 (*P* < 0.001^*^) respectively. At the cut-off value was − 5.85 for miR-320a-5p, the sensitivity and specificity were 75.31% and 74.19%, respectively. While the cut-off value was − 8.68 for miR-15b-5p, the sensitivity and specificity were 70.37% and 64.52%, respectively. Additionally, the cut-off value was 3.07 for miR-19a-3p, the sensitivity and specificity were 86.42% and 58.06%, respectively (Table 3).

**Table 3:** Area under the curve (AUC) (95% confidence interval) for the examined MiRNAs between tested variants

Marker	AUC	95% CI for AUC	*p*-value
miR-320a-5p	0.708	0.598–0.819	0.001^*^
miR-15b-5p	0.700	0.594–0.806	0.001^*^
miR-19a-3p	0.715	0.601–0.829	< 0.001^*^
miR-374b-5p	0.583	0.468–0.699	0.174
miR-423-5p	0.611	0.497–0.724	0.071

### Circulating miRNA signatures in COVID-19 patients’ plasma obtained from (severe and moderate) (males and females)

**Fig. 5:**
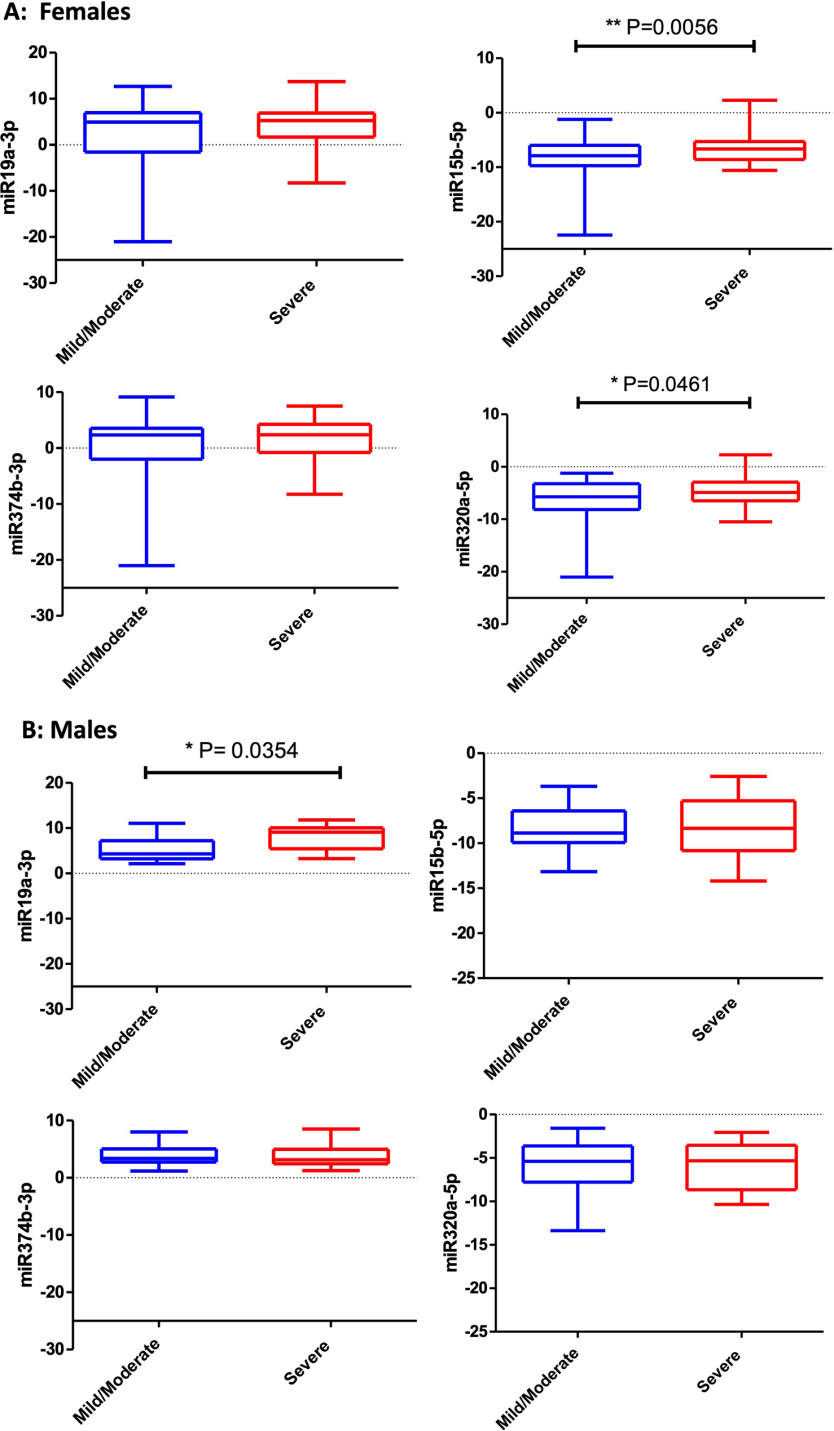
**a**, **b** Differential expression of the five tested miRNAs of SARS-CoV-2-infected patients (according to severity and gender of cases. The cases were 24 mild/moderate males, 30 mild/moderate females, 17 severe males, and 41 severe females, and 112 healthy controls

To determine whether the selected miRNAs could differentiate between the severely and moderately infected male and female patients, we compared the expression of the selected 5 miRNAs from each gender in mild/moderate versus severe infected patients. Interestingly enough, miRNA-320a-5p and miRNAs-15p-5p expressions were statistically downregulated in mild/moderate female cases compared to severe female cases (*p-value*, 0.0461, 0.0056), respectively as depicted in Fig. 5a. In addition to that MIR-19a-p expression in severe male cases was statistically upregulated compared to mild/moderate male cases (*p-value*, 0.00354), as depicted in Fig. 5b. These results shed a light on the importance of these three miRNAs to differentiate between clinical severity of the infected patients in a gender specific way.

### Correlation between

#### The selected miRNAs and various diagnostic lab parameters in all cases

A significant correlation was observed between miR-320a-5p and miR-374b-5p expression and TLC with *p-values* of 0.021 and 0.008, respectively, in the COVID-infected patients. This indicates that TLC levels with the expression level of these miRNAs could be a good indicator for the SARS-CoV-2 viral infection, i.e., the TLC level gets high while miR-320a-5p was downregulated, and miR-374b-5p was upregulated during COVID-19 infection with Spearman coefficient (r_s_ = 0.488), and (r_s_ = 0.548), respectively. A significant correlation was found between miR-15b-5p expression and ALT levels (*p-value* 0.021) in COVID-19 patients. This means that ALT levels get high while miR-15b-5p was downregulated (r_s_ = 0.269). A significant correlation was found between miR-320a-5p expression and K level in the blood, with a *P-value* of 0.005 in COVID-19-infected patients. Indicating that K levels get high while miR-320a-5p is downregulated (r_s_ = 0.333). There is a significant correlation between miR-374b-5p expression and D-Dimer level in the blood of COVID-infected patients with a *P-value* of 0.030, indicating that D-Dimer levels get high while miR-15b-5p was upregulated (r_s_ = 0.463) (Table S4).

#### The selected miRNAs and various diagnostic lab parameters in the 2nd wave (Beta variant)

The results showed a significant correlation between miR-320a-5p and serum K-level, with a *p-value* 0.006 in the beta variant COVID-19 patients. This means that K-levels get high while miR-320a-5p was downregulated (r_s_ = 0.388). Similarly, a significant correlation was detected between miR-19a-3p and K, pH, as well as the serum albumin levels in cases infected with the beta variant, with *p-values* 0.013, 0.038, and 0.036 in COVID-19 patients, respectively. This means that K, pH, and serum albumin levels get high while miR-19a-3p was upregulated with r_s_ = 0.353, 0.346, and 0.297, respectively (Table S5).

#### The selected miRNAs and various diagnostic lab parameters in the 3rd wave (Delta variant)

A significant correlation was found between miR-320a-5p and PLT count, TLC, and INR levels, with *p-values* 0.010, 0.005, and 0.028 in the delta variant COVID-19 patients, respectively. This means that PLT, TLC, and INR levels get high while miR-320a-5p was downregulated with r_s_ = 0.507, 0.943, and 0.468, respectively. In addition, a significant correlation between miR-423-5p and PLT count, TLC, and serum albumin levels was observed with *p-values* of 0.032, 0.042, and 0.020 in the delta variant COVID-19 patients. This means that PLT and TLC levels get high and serum albumin level gets low while miR-423-5p expression levels are not affected greatly by the infection with (r_s_ = 0.431), (r_s_ = 0.829), (r_s_ = 0.482), respectively, and significant correlation between miR-374b-5p and PLT and CRP levels in cases infected with the delta variant with *p-value* 0.002, 0.014 in the delta variant COVID-19 patients. This means that PLT and CRP levels get high by the upregulation of miR-374b-5p (with r_s_ = 0.585, r_s_ = 0.640, respectively (Table S6).

#### The selected miRNAs and various diagnostic lab parameters in the mild/moderate cases

It was found that there is a significant correlation between miR-423-5p expression and TLC and D-dimer levels, with *p-value*s of 0.036 and 0.023 in moderately infected COVID patients, meaning that as the TLC and D-dimer levels get high, while miR-423-5p expression levels were not affected greatly by the infection, with r_s_ = 0.786 and 0.673, respectively. A significant correlation was found between miR-19a-3p and pH and HCO_3_ in moderate cases, with *p-values* of 0.026 and 0.009. These findings imply that the pH and HCO_3_ levels increase by the downregulation of miR-19a-3p with r_s_ = 0.445 and 0.522, alongside complex physiological mechanisms. Moreover, there was a significant correlation between miR-374b-5p and serum albumin and INR in moderate cases. i.e., INR levels get high, and Serum albumin level gets low while miR-374b-5p is downregulated with *p-value*s, 0.047, 0.036, and r_s_ = 0.338, r_s_ = 0.430, respectively (Table S7). The severity analyses (moderate vs. severe) were performed using the combined cohort of both waves (Beta and Delta). This was done to ensure adequate statistical power, as separating severity groups by wave would have resulted in small subgroup sizes.

#### The selected miRNAs and various diagnostic lab parameters in the severe cases

It was found that there is a significant correlation between miR-320a-5p expression and TLC and K levels, with *p-value*s of 0.021 and 0.045 in severely infected COVID-19 patients, meaning that TLC and K levels get high while miR-320a-5p was downregulated (with r_s_ = 0.590 and r_s_ = 0.327, respectively). In addition, a significant correlation between miR-423-5p and PLT and TLC in severe cases with *p-value*s of 0.049 and 0.024 in severely infected COVID patients, meaning that PLT and TLC levels get high while miR-423-5p expression levels were not affected greatly by the infection (with r_s_ = 0.306 and r_s_ = 0.579, respectively. (Table S8).

## Discussion

Managing COVID-19 patients is extremely challenging, especially when it comes to patient stratification, treatment selection, and tracking the disease’s progression. Under this concept, the development of novel, minimally invasive biomarkers with prognostic significance may aid in identifying patients who are more likely to develop severe COVID-19 [46], particularly among older comorbid patients [18]. Biomarkers are essential strategies for tackling these issues and for evaluating the prognosis of the disease [46]. Circulating miRNAs are among the new biomarkers that have drawn a lot of attention because of their dynamic expression profiles in response to viral infections [46–48]. For the discovery of microRNA and its function in post-transcriptional gene control, Victor Ambros and Gary Ruvkun were granted the 2024 Nobel Prize in Physiology or Medicine [49]. This addresses the importance of miRNAs as biomarkers by providing fresh perspectives on the course of the illness and the effectiveness of treatment; circulating miRNA profiling has the potential to greatly enhance the care of COVID-19 patients [50, 51]. The implementation of this technique could result in more accurate treatment plans and a deeper comprehension of the fundamental mechanisms of SARS-CoV-2 infection [50, 52, 53]. The use of peripheral blood RNA analysis in regular contexts has been recognized for the future [54]. The COVID-19 outbreak continues, according to recent reports from the WHO [1], which highlights the critical necessity to develop appropriate methods to effectively cure patients who have major consequences and guarantees the importance of early and precise diagnosis of SARS-CoV-2 infections. Herein, a set of miRNAs was identified whose expression is deregulated during SARS-CoV-2 infection. The NCBI database provided SARS-CoV-2 sequences isolated from nine different countries to figure out the binding of miRNAs [55]. It stated that the seed region and the target sites are the same for the miR-15 family [55]. It was reported that miR-15b-5p plays a direct antiviral role by targeting the RNA-dependent RNA polymerase of SARS-CoV-2, thereby suppressing viral replication. Its downregulation may impair apoptosis and promote viral persistence, contributing to immune evasion and tissue damage [28]. In this study, miR-15b-5p, miR-320a-5p, miR-19a-3p, miR-374b-5p, and miR-423-5p were evaluated. These miRNAs were selected based on biological plausibility and prior mechanistic evidence, not because they are definitively superior to all other reported COVID-19-related miRNAs. Our findings support their diagnostic value (particularly miR-15b-5p and miR-320a-5p), but we do not position them as exclusive or superior prognostic markers. Additional miRNAs reported in the literature may also hold prognostic potential, and comparative multi-marker panels may ultimately provide the strongest clinical utility.

The present study revealed the profound downregulation of miR-15b-5p in all infected cases (severe and moderate) and different variants according to the ROC curve, with AUC 1, sensitivity 100%, and specificity 100%. This is in accordance with Kim et al. on hamster lung tissues infected by SARS-CoV-2: hsa-miR-15b-5p attached to SARS-CoV-2 has a target score of 99 [55]. Numerous miRNAs have been demonstrated or anticipated to target SARS-COV-2 RNAs [56]. It has been discovered that several miRNAs target the RNA S-glycoprotein sequence of SARS-COV-2, which interacts with the angiotensin-converting enzyme II to allow the virus to enter host cells. One of them is has-miR15b-5p, which has been demonstrated to bind to the receptor-binding region of the S gene [57, 58]. Additionally, the miR-15 family targets the antiapoptotic gene B-cell leukemia/lymphoma 2 protein (Bcl-2) to control apoptosis [55, 59, 60]. In the present study, our findings are aligned with Kim’s theory, which states that miR-15b-5p expression was downregulated [55]. As the downregulation of hsa-miR-15b-5p inhibits apoptosis and promotes the growth of infected cells, it may allow SARS-CoV-2 to evade the host immune defense [55]. Contrary to our findings, Bautista-Becerril et al. confirmed the upregulation of miR-15b-5p in severe cases [61]. They related this finding to the inhibition of CD8 + T-cell activation, repressing IL-2 and IFN production, so miR-15b-5p enhanced the viral replication and strengthened illness severity [61]. This may be related to different genetic features of the patients enrolled in this study compared to others, as previously reported [4].

A comparison was conducted to differentiate between different SARS-COV-2 strains (variants) in two different waves, and results revealed no significant change in the expression of miR-15b-5p between different infected patients (delta and beta), thus, it could be a pivotal diagnostic target for all COVID-19 strains. The differential expression (DE) of miR-15b-5p could not differentiate between the tested variants. Interestingly, upon a correlation between the miR-15b-5p expression and Lab parameters of the SARS-CoV-2 infected patients, according to the Spearman coefficient, ALT levels get high while miR-15b-5p is downregulated. This implies that the downregulation of miR-15b-5p may somehow affect the liver function of infected COVID-19 patients. MiR-320a-5p is associated with endothelial dysfunction and vascular injury. Its downregulation correlates with increased thrombotic risk and impaired immune modulation, contributing to complications such as deep vein thrombosis and ARDS [31]. Duecker et al. addressed that in COVID-19 patients with severe respiratory failure, the miR-320a-30 family is significantly downregulated [31]. Regarding inflammation and endothelial dysfunction, the analysis revealed that the members of the miR-320 family regulate 20 genes in the Hippo signaling network [55]. These include 13 genes in the transforming growth factor beta (TGF-β) signaling pathway and 12 genes that control epithelial paracellular permeability and adhesion junctions [31]. Starikova et al. and Jiang et al. have reported that patients with deep vein thrombosis (DVT) have downregulated the miRNA-320 family [62, 63]. Cai et al. mentioned that since DVT is another serious side effect of COVID-19, it is critical to identify high-risk COVID-19 patients so that thrombosis prophylaxis can be started as soon as possible [64]. Moreover, this study has shown a significant downregulation of miR-320a-5p, similar to the earlier research. This is correlated with coagulation and inflammatory markers in individuals with moderate to severe respiratory failure brought on by COVID-19 [18, 31]. Unlike Grehl et al. and Fayyad-Kazan et al., they stated that miR-320a-5p is upregulated as the immune response differentiates moderate from severe cases, involved in the infection response, demonstrating the molecular status of the lung tissue [30, 65]. As of the profound downregulation of this miR-320a-5p in the present study in all infected cases, according to the ROC curve with AUC 0.920, sensitivity 91.96% and specificity 100.0%. A comparison was conducted to differentiate between different SARS-CoV-2 strains (variants) in different COVID-19 waves; there was a significant difference in the expression of miR-320a-5p between the two infected patients (delta and beta). Thus, it could be a pivotal diagnostic target. Interestingly, upon the correlation between the miR-320a-5p expression and Lab parameters of all the SARS-COV-2 infected patients, according to the Spearman coefficient, there was a correlation with TLC and K-level readings. This means that TLC and K levels get high while miR-320a-5p is downregulated. Giving correlation also in the 2nd wave infected patients with K levels. Correlations in 3rd wave cases with PLTs, TLC, and INR levels. These findings greatly support the mechanism of miR-320a-5p in COVID-19 patients. This effective intravascular defense system demonstrates how closely the coagulation and immune systems interact. However, people suffering from severe COVID-19 infection may be at risk for death if it is not brought under control [4]. While ROC analysis was statistically significant, the ROC metrics for differentiating between waves do not meet clinical diagnostic thresholds and should be interpreted as exploratory observations rather than actionable diagnostic tools.

Ivashchenko et al. and Saçar Demirci et al. have mentioned that if the identified miRNAs are expressed in the target host cells, then host miRNAs are relevant for inhibiting viral replication [66, 67]. The findings of the present study are in agreement with M Fayyad-Kazan et al., who found that the plasma of COVID-19 patients exhibited differential expression of eight miRNAs, including miR-19a-3p as the most upregulated [30]. According to reports, Interferon Regulatory Factor 1 (IRF1) is necessary for the M1 macrophage inflammatory response and viral defense [7]. MiR-19a-3p has been shown to reduce inflammation and limit M1 macrophage polarization by inhibiting the (STAT1/IRF1) pathway [29, 68]. Furthermore, miR-19a-3p is one of the miR-17–92 cluster’s key players in cell division and proliferation, inflammation, immunology, and immunological processes [69–71]. The miR-19a-3p upregulation of the TGF-β signaling pathway was the target, actions that inhibit the immune system and reduce inflammation [30, 51, 72]. In the present study, the DE of miR-19a-3p could differentiate between the tested variants. Interestingly, our findings revealed a significant correlation between the miR-19a-3p expression and Lab parameters of 2nd wave cases of the SARS-CoV-2 infected patients, according to the Spearman coefficient, a correlation with K, pH, and serum albumin readings. This means that K, pH, and serum albumin levels get high when miR-19a-3p is upregulated. Giving a correlation also in mild/ moderate infected patients with pH and HCO_3_ levels. MiR-374b-5p: Involved in T-cell regulation and cytokine signaling. It also inhibits the C-FLIP protein, which affects viral protein expression and apoptosis, influencing immune balance and viral clearance [32]. According to Qi et al., the long non-coding RNA nuclear paraspeckle assembly transcript 1 (lncRNA NEAT1) may affect the host immune response through two miRNAs, hsa-miR-374-5p and hsa-miR-155-5p, which regulate the expression of various inflammatory mediators [12]. It is still essential to identify the precise mechanisms for the association of miR-374b-5p upregulation and host immune response in COVID-19 patients [12]. In contrast to the findings of the current investigation, Srivastava et al. reported that their study groups shared ten miRNAs that were markedly upregulated, particularly hsa-miR-374b-5p, and two miRNAs, hsa-miR-320a-5p [73]. These findings are in contrast to the present work, where miR-320a-5p is downregulated, and miR-374b-5p is upregulated. The DE of miR-374b-5p could differentiate between the tested variants. Interestingly, upon the correlation between the miR-374b-5p expression and Lab parameters of all infected cases according to the Spearman coefficient, there was a correlation with TLC and D-dimer readings. This means that TLC and D-dimer levels get high with miR-374b-5p being upregulated. There is also a correlation in 3rd wave cases of the SARS-COV-2 infected patients, according to the Spearman coefficient, with PLT and CRP readings. This means that PLT and CRP levels get high with miR-374b-5p is upregulated. Correlation was also found in mild/moderate infected patients with serum albumin and INR levels. This means that serum albumin levels get low when miR-374b-5p is upregulated, and INR levels get high when miR-374b-5p is upregulated. MiR-423-5p is linked to cardiac stress and hypoxia. Previous studies suggest its involvement in cardiovascular complications seen in severe COVID-19 cases [33]. Farr et al. discovered that a 3-miRNA signature (miR-423-5p, miR-23a-3p, and miR-195-5p) could independently classify COVID-19 cases with a 99.9% accuracy rate. miR-423-5p upregulation [33]. The findings of the present study led to conflicts with Farr and his colleagues since, in the Egyptian population, miR-423-5p DE has not changed in the diseased group from the control group. Interestingly, upon the correlation between the miR-423-5p expression and Lab parameters of all infected cases according to the Spearman coefficient, there was a correlation with TLC readings (r_s_ = 0.536^*^). There is also a correlation in 3rd wave cases of the SARS-COV-2 infected patients with PLT, TLC, and serum albumin readings. This means that PLT and TLC levels get high with miR-423-5p DE in 3rd wave cases, respectively, and Serum albumin levels get Low with (r_s_ = -0.482^*^). There is also a correlation in Mild/ Moderate infected patients with TLC levels (r_s_ = 0.786^*^). This means that TLC levels get very high with miR-423-5p in mild/moderate cases. Also, there was a correlation in severely infected patients with PLT and TLC levels. Collectively, according to the above findings, certain miRNAs that directly bind to the SARS-CoV-2 genome, such as hsa-miR-15b-5p, miR-320a-5p, miR-19a-3p, and miR-374b-5p, may have a significant role in SARS-CoV-2 infection and may even serve as diagnostic biomarkers for the virus.

## Conclusions

As the SARS-CoV-2 virus continues to mutate, the need for innovative and effective biomarkers, such as circulating miRNAs, becomes increasingly imperative for accurate diagnosis and disease monitoring. The discovery of upregulated, downregulated, and unchanged miRNAs, along with their correlation with clinical parameters, underscores their value as potential biomarkers for disease severity and progression. The data presented here delves into the unique landscape of circulating miRNAs in COVID-19 patients, shedding light on their potential as non-invasive diagnostic tools. The study’s findings, particularly the consistent miRNA expression patterns across different variants and severity levels, emphasize the reliability and diagnostic potential of miRNAs in COVID-19 management. Our findings underscore the crucial role of miRNAs as potential biomarkers in the diagnosis and monitoring of COVID-19, offering new avenues for disease detection and control in the face of evolving viral variants. The study’s findings pave the way for further exploration of miRNAs as powerful tools in the fight against infectious diseases like COVID-19. However, future studies incorporating transcriptome-wide miRNA profiling are recommended to provide a more comprehensive and unbiased representation.

## Supplementary Information

Below is the link to the electronic supplementary material.

Supplementary Material 1

## Data Availability

All data generated or analyzed during this study are included in this main manuscript and supplementary file.
