# Supplementary material for: Host MiRNA responses during different waves of SARS-CoV-2: diagnostic implications of miR-19a-3p, miR-374b-5p, miR-15b-5p, and miR-320a-5p expression
Source: BMC Infect Dis. 2026 Feb 16;26:662. doi: 10.1186/s12879-026-12706-y (PMC13032573; doi:10.1186/s12879-026-12706-y)
Supplement: Supplementary file 1 — Supplementary Material 1 [file 12879_2026_12706_MOESM1_ESM.docx]

**Table S1.** Descriptive analysis of the studied cases according to laboratory investigations in the case group

| **Lab parameter** | **N** | **Min. – Max.** | **Mean ± SD.** | **Median (IQR)** |
| --- | --- | --- | --- | --- |
| **CBC** |  |  |  |  |
| **Hb** | **82** | 5.90 – 19.50 | 11.87 ± 2.30 | 11.70(10.70 –13.20) |
| **Hematocrit** | **70** | 15.80 – 50.10 | 35.88 ± 6.28 | 35.50 (33.0 – 39.80) |
| **PLT** | **80** | 74.0 – 530.0 | 266.2 ± 106.4 | 259.0(174.0 –338.0) |
| **TLC** | **22** | 1.70 – 23.30 | 10.94 ± 5.43 | 9.50 (7.80 – 15.50) |
| **Renal function** |  |  |  |  |
| **Urea** | **68** | 19.0 – 286.0 | 73.35 ± 54.54 | 55.0 (39.0 – 90.0) |
| **Creatinine** | **76** | 0.20 – 9.30 | 1.26 ± 1.72 | 0.64 (0.50 – 1.05) |
| **Electrolytes** |  |  |  |  |
| **Na** | **68** | 110.0 – 193.0 | 138.2 ± 10.53 | 138.0(133.0 –141.0) |
| **K** | **69** | 2.40 – 7.0 | 4.30 ± 0.94 | 4.30 (3.70 – 4.70) |
| **Arterial blood gas** |  |  |  |  |
| **PH** | **54** | 7.23 – 7.54 | 7.43 ± 0.07 | 7.43 (7.40 – 7.49) |
| **PCO_2_** | **55** | 11.0 – 82.0 | 37.75 ± 11.58 | 35.0(31.45 –43.50) |
| **HCO_3_** | **54** | 8.0 – 50.80 | 26.13 ± 7.79 | 25.55(21.60 –29.90) |
| **PO_2_** | **50** | 20.0 – 157.0 | 64.01 ± 31.49 | 57.0 (39.0 – 80.0) |
| **Liver enzymes** |  |  |  |  |
| **T. Bilirubin** | **70** | 0.10 – 1.70 | 0.66 ± 0.27 | 0.60 (0.50 – 0.80) |
| **S. Albumin** | **73** | 2.0 – 40.0 | 3.78 ± 4.33 | 3.40 (3.0 – 3.60) |
| **ALT** | **66** | 12.0 – 750.0 | 54.79 ± 96.71 | 30.50 (21.0 – 47.0) |
| **AST** | **69** | 13.0 – 422.0 | 48.32 ± 55.21 | 33.0 (22.0 – 48.0) |
| **Inflammatory markers** |  |  |  |  |
| **INR** | **56** | 1.0 – 4.40 | 1.25 ± 0.56 | 1.06 (1.0 – 1.24) |
| **CRP** | **22** | 6.0 – 96.0 | 60.82 ± 32.30 | 48.0 (48.0 – 96.0) |
| **D-Dimer** | **22** | 0.49 – 10.0 | 1.99 ± 2.08 | 1.19 (1.09 – 2.09) |

IQR: Inter quartile range; N: Number; SD: Standard deviation

**Table S2.** Comparison between the two studied groups according to different LogFC miRNA

| **LogFC** | **Cases (n = 112)** | **Control (n = 112)** | **U** | **p** |
| --- | --- | --- | --- | --- |
| miR-320a-5p |  |  |  |  |
| Min. – Max. | -21.01 – 3.60 | 0.0 – 0.0 | 1008.00^*^ | <0.001^*^ |
| Mean ± SD. | -5.0 ± 3.65 | 0.0 ± 0.0 |  |  |
| Median (IQR) | -4.88(-6.91 – -2.91) | 0.0 (0.0 – 0.0) |  |  |
| miR-15b-5p |  |  |  |  |
| Min. – Max. | -22.45 – -0.09 | 0.0 – 0.0 | 0.000^*^ | <0.001^*^ |
| Mean ± SD. | -7.74 ± 3.07 | 0.0 ± 0.0 |  |  |
| Median (IQR) | -7.96(-9.73 – -6.02) | 0.0 (0.0 – 0.0) |  |  |
| miR-423-5p |  |  |  |  |
| Min. – Max. | -17.13 – 8.35 | 0.0 – 0.0 | 6048.00 | 0.621 |
| Mean ± SD. | -0.16 ± 2.76 | 0.0 ± 0.0 |  |  |
| Median (IQR) | -0.09(-1.62 – 1.39) | 0.0 (0.0 – 0.0) |  |  |
| miR-19a-3p |  |  |  |  |
| Min. – Max. | -9.48 – 13.71 | 0.0 – 0.0 | 896.00^*^ | <0.001^*^ |
| Mean ± SD. | 5.13 ± 3.81 | 0.0 ± 0.0 |  |  |
| Median (IQR) | 5.50 (3.05 – 7.64) | 0.0 (0.0 – 0.0) |  |  |
| miR-374b-5p |  |  |  |  |
| Min. – Max. | -13.53 – 9.14 | 0.0 – 0.0 | 2128.00^*^ | <0.001^*^ |
| Mean ± SD. | 2.20 ± 2.95 | 0.0 ± 0.0 |  |  |
| Median (IQR) | 2.45 (0.60 – 3.98) | 0.0 (0.0 – 0.0) |  |  |

IQR: Inter quartile range; SD: Standard deviation; U: Mann Whitney test; p: *p-value* for comparing between the two studied groups; *: Statistically significant at p ≤ 0.05

**Table S3.** Comparison between the two studied subgroups of COVID-19 Waves according to different LogFC miRNA

| **LogFC** | **COVID Wave** | | **U** | **P** |
| --- | --- | --- | --- | --- |
|  | **2^nd^ wave (n = 81)** | **3^rd^ wave (n = 31)** |  |  |
| **miR320** |  |  |  |  |
| Min. – Max. | -21.01 – 3.60 | -11.71 – 1.62 | 732.00^*^ | 0.001^*^ |
| Mean ± SD. | -4.44 ± 3.71 | -6.47 ± 3.09 |  |  |
| Median (IQR) | -4.25 (-5.70 – -2.70) | -6.80 (-8.44 – -5.50) |  |  |
| **miR15b** |  |  |  |  |
| Min. – Max. | -22.45 – -0.09 | -14.20 – -1.30 | 752.50^*^ | 0.001^*^ |
| Mean ± SD. | -7.29 ± 3.19 | -8.91 ± 2.39 |  |  |
| Median (IQR) | -7.06 (-9.21 – -5.59) | -9.02(-10.40 – -8.04) |  |  |
| **miR423** |  |  |  |  |
| Min. – Max. | -17.13 – 8.35 | -5.87 – 5.79 | 977.50 | 0.071 |
| Mean ± SD. | -0.002 ± 2.96 | -0.59 ± 2.12 |  |  |
| Median (IQR) | 0.19 (-1.45 – 1.56) | -0.83 (-1.81 – 0.63) |  |  |
| **miR19a** |  |  |  |  |
| Min. – Max. | -9.48 – 13.71 | -3.29 – 11.05 | 716.00^*^ | <0.001^*^ |
| Mean ± SD. | 5.84 ± 3.67 | 3.29 ± 3.58 |  |  |
| Median (IQR) | 5.89 (3.68 – 8.23) | 2.58 (0.63 – 5.93) |  |  |
| **miR374b** |  |  |  |  |
| Min. – Max. | -13.53 – 9.14 | -1.21 – 8.02 | 1046.50 | 0.174 |
| Mean ± SD. | 1.94 ± 3.12 | 2.88 ± 2.39 |  |  |
| Median (IQR) | 2.15 (0.13 – 3.68) | 2.84 (0.86 – 4.57) |  |  |

IQR: Inter quartile range; SD: Standard deviation ; H: H for Kruskal Wallis test, Pairwise comparison bet. each 2 groups was done using Post Hoc Test (Dunn's for multiple comparisons test); p: p-value for comparing between three studied subgroups; p_1_: p-value for comparing between 2^nd^ wave and 3^rd^ wave; p_2_: p-value for comparing between 2^nd^ wave and Control; p_3_: p-value for comparing between 3^rd^ wave and Control; *: Statistically significant at p ≤ 0.05

**Table S4.** Correlation between different LogFC with laboratory investigations in all cases

|  | **miR320a-5p** | | **miR-15b-5p** | | **miR-423-5p** | | **miR-19a-3p** | | **miR-374b-5p** | |
| --- | --- | --- | --- | --- | --- | --- | --- | --- | --- | --- |
|  | **r_s_** | **P** | **r_s_** | **p** | **r_s_** | **p** | **r_s_** | **P** | **r_s_** | **p** |
| Age (years) | -0.050 | 0.599 | -0.018 | 0.853 | 0.078 | 0.415 | 0.083 | 0.383 | 0.154 | 0.104 |
| Hb | 0.035 | 0.758 | 0.000 | 0.998 | -0.061 | 0.586 | 0.005 | 0.963 | -0.042 | 0.705 |
| Hematocrit | 0.031 | 0.799 | 0.022 | 0.858 | 0.030 | 0.806 | 0.026 | 0.833 | 0.116 | 0.338 |
| PLT | 0.059 | 0.603 | 0.057 | 0.619 | 0.105 | 0.353 | 0.118 | 0.299 | 0.216 | 0.054 |
| TLC | 0.488^*^ | 0.021^*^ | 0.194 | 0.386 | 0.536^*^ | 0.010^*^ | 0.150 | 0.506 | 0.548^*^ | 0.008^*^ |
| Urea | 0.207 | 0.090 | -0.027 | 0.842 | 0.028 | 0.840 | -0.110 | 0.418 | 0.151 | 0.266 |
| Creatinine | 0.091 | 0.435 | 0.090 | 0.464 | 0.000 | 0.997 | 0.069 | 0.579 | 0.062 | 0.616 |
| Na | -0.060 | 0.625 | -0.018 | 0.881 | -0.050 | 0.669 | -0.088 | 0.450 | 0.088 | 0.451 |
| K | 0.333^*^ | 0.005^*^ | 0.118 | 0.339 | -0.014 | 0.911 | -0.009 | 0.943 | -0.005 | 0.967 |
| pH | 0.104 | 0.453 | 0.145 | 0.233 | 0.073 | 0.554 | 0.226 | 0.062 | 0.082 | 0.502 |
| PCO_2_ | -0.032 | 0.819 | 0.100 | 0.470 | 0.026 | 0.854 | 0.212 | 0.124 | 0.123 | 0.375 |
| HCO_3_ | -0.027 | 0.845 | 0.037 | 0.790 | 0.113 | 0.410 | 0.037 | 0.790 | -0.104 | 0.449 |
| PO_2_ | 0.110 | 0.448 | 0.111 | 0.425 | 0.215 | 0.119 | 0.137 | 0.323 | -0.036 | 0.795 |
| Total bilirubin | -0.009 | 0.944 | -0.090 | 0.534 | -0.080 | 0.582 | -0.014 | 0.921 | 0.178 | 0.217 |
| Serum albumin | 0.169 | 0.153 | -0.132 | 0.275 | 0.018 | 0.879 | -0.029 | 0.810 | 0.066 | 0.588 |
| ALT | 0.113 | 0.365 | 0.269^*^ | 0.021^*^ | -0.042 | 0.724 | 0.216 | 0.066 | -0.222 | 0.059 |
| AST | 0.003 | 0.978 | 0.016 | 0.897 | -0.097 | 0.438 | 0.075 | 0.551 | -0.086 | 0.493 |
| INR | -0.141 | 0.300 | -0.159 | 0.193 | 0.014 | 0.912 | -0.004 | 0.974 | -0.159 | 0.192 |
| CRP | 0.302 | 0.171 | 0.010 | 0.964 | 0.038 | 0.865 | 0.263 | 0.237 | 0.138 | 0.540 |
| D-Dimer | -0.021 | 0.924 | 0.080 | 0.725 | 0.346 | 0.115 | 0.175 | 0.435 | 0.463^*^ | 0.030^*^ |

r_s_: Spearman coefficient

*: Statistically significant at p ≤ 0.05

**Table S5.** Correlation between different LogFC with laboratory investigations in 2^nd^ COVID wave cases (beta variant)

|  | **miR320a-5p** | | **miR-15b-5p** | | **miR-423-5p** | | **miR-19a-3p** | | **miR-374b-5p** | |
| --- | --- | --- | --- | --- | --- | --- | --- | --- | --- | --- |
|  | **r_s_** | **P** | **r_s_** | **p** | **r_s_** | **p** | **r_s_** | **P** | **r_s_** | **P** |
| Age (years) | -0.093 | 0.410 | -0.027 | 0.811 | 0.063 | 0.576 | 0.032 | 0.779 | 0.099 | 0.379 |
| Hb | -0.009 | 0.945 | -0.057 | 0.678 | -0.062 | 0.650 | -0.053 | 0.699 | 0.086 | 0.528 |
| Hematocrit | 0.034 | 0.821 | 0.000 | 0.999 | 0.037 | 0.808 | -0.036 | 0.812 | 0.160 | 0.288 |
| PLT | -0.072 | 0.600 | 0.060 | 0.662 | -0.007 | 0.962 | 0.064 | 0.644 | 0.081 | 0.559 |
| TLC | 0.340 | 0.197 | 0.230 | 0.392 | 0.437 | 0.090 | 0.189 | 0.484 | 0.476 | 0.063 |
| Urea | 0.220 | 0.146 | 0.063 | 0.681 | -0.026 | 0.866 | 0.042 | 0.785 | 0.018 | 0.907 |
| Creatinine | 0.142 | 0.322 | 0.005 | 0.972 | -0.102 | 0.477 | -0.087 | 0.544 | -0.066 | 0.643 |
| Na | -0.041 | 0.781 | 0.070 | 0.631 | 0.026 | 0.857 | -0.021 | 0.887 | -0.085 | 0.562 |
| K | 0.388^*^ | 0.006^*^ | 0.223 | 0.123 | 0.043 | 0.767 | 0.353^*^ | 0.013^*^ | 0.158 | 0.279 |
| pH | 0.188 | 0.271 | 0.116 | 0.500 | 0.047 | 0.786 | 0.346^*^ | 0.038^*^ | 0.275 | 0.105 |
| PCO_2_ | -0.047 | 0.782 | 0.123 | 0.469 | 0.068 | 0.690 | 0.106 | 0.534 | 0.053 | 0.755 |
| HCO_3_ | -0.048 | 0.780 | 0.136 | 0.430 | 0.236 | 0.166 | 0.193 | 0.260 | 0.209 | 0.222 |
| PO_2_ | 0.050 | 0.785 | -0.216 | 0.235 | -0.105 | 0.566 | -0.222 | 0.221 | 0.160 | 0.381 |
| Total bilirubin | -0.033 | 0.823 | -0.193 | 0.189 | -0.052 | 0.724 | -0.082 | 0.580 | 0.125 | 0.397 |
| Serum albumin | 0.102 | 0.480 | 0.270 | 0.058 | 0.025 | 0.861 | 0.297^*^ | 0.036^*^ | -0.108 | 0.455 |
| ALT | 0.078 | 0.604 | -0.030 | 0.843 | -0.162 | 0.276 | 0.053 | 0.725 | -0.179 | 0.228 |
| AST | -0.077 | 0.609 | -0.227 | 0.124 | -0.038 | 0.800 | -0.122 | 0.415 | -0.252 | 0.087 |
| INR | -0.177 | 0.317 | -0.086 | 0.630 | 0.010 | 0.956 | -0.189 | 0.284 | -0.134 | 0.450 |
| CRP | 0.179 | 0.672 | -0.495 | 0.212 | -0.481 | 0.227 | -0.591 | 0.123 | 0.000 | 1.000 |
| D-Dimer | 0.190 | 0.651 | 0.095 | 0.823 | 0.524 | 0.183 | 0.060 | 0.888 | 0.452 | 0.260 |

r_s_: Spearman coefficient

*: Statistically significant at p ≤ 0.05

**Table S6.** Correlation between different LogFC with laboratory investigations in 3^rd^ COVID wave cases (delta variant)

|  | **miR320a-5p** | | **miR-15b-5p** | | **miR-423-5p** | | **miR-19a-3p** | | **miR-374b-5p** | |
| --- | --- | --- | --- | --- | --- | --- | --- | --- | --- | --- |
|  | **r_s_** | **P** | **r_s_** | **p** | **r_s_** | **P** | **r_s_** | **P** | **r_s_** | **P** |
| Age (years) | -0.072 | 0.698 | -0.085 | 0.649 | 0.078 | 0.678 | 0.209 | 0.260 | 0.291 | 0.112 |
| Hb | 0.050 | 0.809 | 0.087 | 0.674 | -0.151 | 0.462 | -0.051 | 0.806 | -0.211 | 0.302 |
| Hematocrit | 0.001 | 0.995 | -0.011 | 0.960 | -0.031 | 0.884 | 0.063 | 0.770 | 0.079 | 0.713 |
| PLT | 0.507^*^ | 0.010^*^ | 0.163 | 0.436 | 0.431^*^ | 0.032^*^ | 0.344 | 0.092 | 0.585^*^ | 0.002^*^ |
| TLC | 0.943^*^ | 0.005^*^ | 0.371 | 0.468 | 0.829^*^ | 0.042^*^ | 0.086 | 0.872 | 0.657 | 0.156 |
| Urea | 0.200 | 0.360 | 0.153 | 0.485 | 0.122 | 0.579 | 0.162 | 0.461 | 0.294 | 0.174 |
| Creatinine | -0.034 | 0.872 | -0.054 | 0.799 | 0.048 | 0.821 | -0.023 | 0.914 | 0.364 | 0.074 |
| Na | 0.015 | 0.951 | 0.261 | 0.280 | -0.010 | 0.968 | -0.087 | 0.724 | 0.104 | 0.673 |
| K | 0.013 | 0.957 | -0.180 | 0.448 | -0.008 | 0.975 | -0.161 | 0.498 | -0.029 | 0.902 |
| pH | 0.091 | 0.719 | 0.071 | 0.779 | -0.052 | 0.837 | 0.037 | 0.885 | -0.137 | 0.588 |
| PCO_2_ | -0.370 | 0.131 | -0.181 | 0.473 | 0.071 | 0.779 | -0.134 | 0.595 | -0.226 | 0.367 |
| HCO_3_ | -0.222 | 0.376 | 0.024 | 0.925 | -0.020 | 0.938 | -0.045 | 0.858 | -0.311 | 0.209 |
| PO_2_ | 0.303 | 0.222 | 0.247 | 0.323 | 0.194 | 0.440 | 0.351 | 0.153 | 0.265 | 0.287 |
| Total bilirubin | 0.050 | 0.826 | -0.058 | 0.796 | 0.208 | 0.353 | 0.032 | 0.888 | -0.045 | 0.842 |
| Serum albumin | 0.131 | 0.552 | 0.077 | 0.728 | -0.482^*^ | 0.020^*^ | -0.210 | 0.337 | -0.364 | 0.087 |
| ALT | 0.028 | 0.909 | 0.009 | 0.972 | 0.151 | 0.537 | -0.014 | 0.955 | 0.233 | 0.336 |
| AST | 0.334 | 0.128 | -0.147 | 0.515 | 0.208 | 0.354 | 0.319 | 0.149 | 0.105 | 0.641 |
| INR | 0.468^*^ | 0.028^*^ | 0.366 | 0.094 | 0.264 | 0.235 | 0.182 | 0.417 | 0.290 | 0.191 |
| CRP | 0.382 | 0.178 | 0.188 | 0.519 | 0.523 | 0.055 | 0.454 | 0.103 | 0.640^*^ | 0.014^*^ |
| D-Dimer | -0.007 | 0.982 | 0.055 | 0.852 | 0.174 | 0.553 | 0.319 | 0.267 | 0.292 | 0.311 |

**r_s_: Spearman coefficient**

*: Statistically significant at p ≤ 0.05

**Table S7**. Correlation between different LogFC with laboratory investigations in Mild/ Moderate cases

|  | **miR320a-5p** | | **miR-15b-5p** | | **miR-423-5p** | | **miR-19a-3p** | | **miR-374b-5p** | |
| --- | --- | --- | --- | --- | --- | --- | --- | --- | --- | --- |
|  | **r_s_** | **P** | **r_s_** | **p** | **r_s_** | **p** | **r_s_** | **P** | **r_s_** | **P** |
| Age (years) | -0.085 | 0.542 | -0.062 | 0.658 | 0.165 | 0.235 | -0.011 | 0.938 | 0.102 | 0.462 |
| Hb | 0.083 | 0.617 | 0.184 | 0.262 | -0.034 | 0.835 | 0.148 | 0.370 | 0.019 | 0.908 |
| Hematocrit | 0.023 | 0.897 | 0.103 | 0.558 | -0.038 | 0.828 | 0.054 | 0.758 | 0.130 | 0.456 |
| PLT | 0.091 | 0.586 | -0.013 | 0.937 | -0.090 | 0.589 | 0.038 | 0.821 | 0.144 | 0.388 |
| TLC | 0.000 | 1.000 | -0.321 | 0.482 | 0.786^*^ | 0.036^*^ | -0.250 | 0.589 | 0.714 | 0.071 |
| Urea | 0.231 | 0.189 | 0.111 | 0.532 | 0.026 | 0.885 | -0.087 | 0.625 | 0.047 | 0.791 |
| Creatinine | 0.112 | 0.517 | -0.087 | 0.614 | -0.055 | 0.751 | -0.203 | 0.235 | 0.145 | 0.398 |
| Na | -0.275 | 0.141 | 0.074 | 0.696 | -0.129 | 0.497 | 0.006 | 0.977 | 0.133 | 0.484 |
| K | 0.322 | 0.077 | 0.160 | 0.389 | -0.014 | 0.942 | 0.279 | 0.128 | 0.071 | 0.706 |
| pH | 0.108 | 0.606 | 0.192 | 0.357 | 0.087 | 0.678 | 0.445^*^ | 0.026^*^ | 0.028 | 0.896 |
| PCO_2_ | 0.011 | 0.958 | 0.086 | 0.682 | 0.167 | 0.425 | 0.244 | 0.240 | 0.169 | 0.420 |
| HCO_3_ | -0.083 | 0.698 | 0.264 | 0.212 | 0.309 | 0.142 | 0.522^*^ | 0.009^*^ | 0.159 | 0.458 |
| PO_2_ | 0.203 | 0.354 | 0.051 | 0.816 | 0.102 | 0.644 | -0.042 | 0.847 | -0.040 | 0.858 |
| Total bilirubin | 0.080 | 0.648 | -0.048 | 0.785 | -0.024 | 0.893 | 0.102 | 0.559 | 0.074 | 0.671 |
| Serum lbumin | 0.232 | 0.179 | 0.229 | 0.186 | -0.141 | 0.420 | 0.319 | 0.062 | -0.338^*^ | 0.047^*^ |
| ALT | 0.181 | 0.348 | 0.140 | 0.469 | -0.124 | 0.523 | 0.293 | 0.123 | -0.147 | 0.448 |
| AST | 0.036 | 0.842 | -0.136 | 0.450 | 0.002 | 0.992 | 0.118 | 0.512 | -0.062 | 0.731 |
| INR | -0.121 | 0.575 | 0.100 | 0.641 | 0.168 | 0.433 | -0.138 | 0.521 | 0.430^*^ | 0.036^*^ |
| CRP | 0.361 | 0.306 | -0.059 | 0.871 | 0.098 | 0.787 | 0.465 | 0.175 | 0.177 | 0.625 |
| D-Dimer | -0.400 | 0.223 | 0.064 | 0.853 | 0.673^*^ | 0.023^*^ | 0.073 | 0.832 | 0.327 | 0.326 |

**r_s_: Spearman coefficient**

*: Statistically significant at p ≤ 0.05

**Table S8.** Correlation between different LogFC with laboratory investigations in severe cases

|  | **miR320a-5p** | | **miR-15b-5p** | | **miR-423-5p** | | **miR-19a-3p** | | **miR-374b-5p** | |
| --- | --- | --- | --- | --- | --- | --- | --- | --- | --- | --- |
|  | **r_s_** | **P** | **r_s_** | **p** | **r_s_** | **p** | **r_s_** | **P** | **r_s_** | **P** |
| Age (years) | 0.015 | 0.914 | 0.067 | 0.618 | 0.001 | 0.992 | 0.220 | 0.097 | 0.193 | 0.147 |
| Hb | -0.028 | 0.860 | -0.169 | 0.278 | -0.100 | 0.522 | -0.146 | 0.351 | -0.085 | 0.589 |
| Hematocrit | 0.115 | 0.510 | -0.039 | 0.823 | 0.119 | 0.496 | 0.012 | 0.947 | 0.079 | 0.650 |
| PLT | 0.050 | 0.754 | 0.101 | 0.526 | 0.306^*^ | 0.049^*^ | 0.180 | 0.254 | 0.234 | 0.137 |
| TLC | 0.590^*^ | 0.021^*^ | 0.095 | 0.737 | 0.579^*^ | 0.024^*^ | 0.293 | 0.289 | 0.372 | 0.172 |
| Urea | 0.206 | 0.242 | 0.151 | 0.394 | -0.029 | 0.869 | 0.257 | 0.143 | 0.131 | 0.461 |
| Creatinine | 0.114 | 0.485 | 0.129 | 0.426 | -0.007 | 0.964 | 0.067 | 0.683 | 0.034 | 0.837 |
| Na | 0.037 | 0.826 | 0.119 | 0.478 | 0.085 | 0.611 | 0.019 | 0.908 | -0.034 | 0.837 |
| K | 0.327^*^ | 0.045^*^ | 0.141 | 0.400 | 0.161 | 0.335 | 0.169 | 0.311 | 0.133 | 0.426 |
| pH | 0.075 | 0.700 | -0.028 | 0.885 | -0.136 | 0.481 | -0.074 | 0.703 | 0.163 | 0.399 |
| PCO_2_ | -0.092 | 0.630 | 0.041 | 0.832 | 0.166 | 0.381 | -0.076 | 0.692 | -0.192 | 0.308 |
| HCO_3_ | -0.022 | 0.907 | 0.013 | 0.947 | 0.198 | 0.293 | -0.103 | 0.590 | -0.079 | 0.678 |
| PO_2_ | 0.061 | 0.763 | -0.234 | 0.241 | -0.257 | 0.196 | 0.010 | 0.960 | 0.377 | 0.053 |
| Total bilirubin | -0.150 | 0.391 | -0.260 | 0.132 | 0.032 | 0.856 | -0.227 | 0.191 | 0.095 | 0.585 |
| Serum albumin | 0.106 | 0.525 | 0.291 | 0.076 | 0.136 | 0.416 | 0.124 | 0.459 | -0.025 | 0.882 |
| ALT | 0.010 | 0.955 | -0.121 | 0.477 | -0.090 | 0.597 | -0.082 | 0.631 | -0.086 | 0.611 |
| AST | 0.005 | 0.977 | -0.118 | 0.494 | 0.032 | 0.851 | -0.115 | 0.504 | -0.202 | 0.238 |
| INR | -0.160 | 0.382 | -0.151 | 0.410 | -0.086 | 0.639 | -0.050 | 0.784 | -0.078 | 0.669 |
| CRP | 0.174 | 0.589 | 0.133 | 0.680 | -0.007 | 0.982 | 0.170 | 0.597 | 0.133 | 0.680 |
| D-Dimer | -0.128 | 0.709 | 0.087 | 0.800 | 0.050 | 0.884 | 0.342 | 0.304 | 0.588 | 0.057 |

**r_s_: Spearman coefficient**

*: Statistically significant at p ≤ 0.05
